# Supplementary material for: Proprioceptive and cutaneous sensations in humans elicited by intracortical microstimulation
Source: eLife. 2018 Apr 10;7:e32904. doi: 10.7554/eLife.32904 (PMC5896877; doi:10.7554/eLife.32904)
Supplement: Source code 1. [file elife-32904-code1.zip › v2_32904R1_source code file 1/stimulation_commands.docx]

**Stimulation commands**

Due to the risks involved in stimulating human cortex, the provided code does not include the ability to interact with the Blackrock Stimulator hardware. Rather, the code is intended to illustrate the communication layer used in our experiments to send stimulation commands.

| Command type | Command prefix (*x*) | Parameter values (*valN*) | Examples | Description |
| --- | --- | --- | --- | --- |
| Waveform  *(Required)* | w | waveformID, polarity, num_pulses, amp_phase1, amp_phase2, pulse_width1, pulse_width2, frequency, interphase_time | *w:1,1,1,100,100,300,300,20,100;*  Sets *waveformID* 1 to CATHODIC leading, single pulse, at 100 uA/phase, 300 us/phase, 20 Hz, and 100 us between phases. | Set waveform parameters |
| Sequence  *(Required)* | q | electrode_num, waveformID | *q:10,1,11,1;*  Assigns *waveformID* 1 to electrodes 10 and 11. | Set electrode(s) for the specified waveformID |
| Stimulation time  *(Optional)* | a | stim_duration | *a:1.5;*  Sets all sequence durations to 1.5 seconds. | Set stimulation time in seconds (default 6). |
| Start stimulation | s | NA | *s;*  Start the stimulation sequence. | Execute the current sequence. |
| Stop stimulation | t | NA | *t;*  Stop the stimulation sequence. | Stop stimulation and reset stimulation time to 6 s. |
| Exit Stimulator | x | NA | *x;*  Exit the stimulation server. | Stop any ongoing stimulation and close communication ports. |

Table 1. Stimulation commands prefix, parameters and examples accepted by StimulationServer. All commands must be terminated with a semicolon.
